# Prevalence of CagA and antimicrobial sensitivity of *H. pylori* isolates of patients with gastric cancer in Egypt

**DOI:** 10.1186/s13027-018-0198-1

**Published:** 2018-07-16

**Authors:** Doaa M. Al-Eraky, Omneya M. Helmy, Yasser M. Ragab, Zeinab Abdul-Khalek, Eman A. El-Seidi, Mohammed A. Ramadan

**Affiliations:** 1 Department of Microbiology and Immunology, October University for Modern Sciences and Arts, Cairo, Egypt; 2 Department of Microbiology and Immunology, Faculty of Pharmacy, Cairo University, Cairo, Egypt; 3 Department of Medical Microbiology and Immunology, Faculty of Medicine, Cairo University, Cairo, Egypt

**Keywords:** *H. pylori*, CagA, Antimicrobial sensitivity, Gastric cancer, Egypt

## Abstract

**Background:**

*Helicobacter pylori (H. pylori*) infection has been recognized as a significant threat for gastric cancer. However, studies that investigated the oncogenic factors and antimicrobial resistance of *H. pylori* in Egyptian isolates with gastric cancer are rare. The current study aimed to examine: (1) The pattern of antimicrobial resistance of *H. pylori* isolates of Egyptian gastric cancer patients, and (2) the prevalence of Cytotoxin-associated gene A (CagA).

**Methods:**

Samples were collected from patients with gastric cancer. Isolation of *H. pylori* was performed using Columbia blood agar supplemented with 10% horse blood, and selective supplement of *H. pylori* for 3 to 5 days at 37 °C under microaerophilic condition. Isolates were identified by biochemical traits of *H. pylori:* oxidase, urease and catalase tests. Antimicrobial susceptibility of *H. pylori* isolates was examined against five antimicrobial agents using disc diffusion method. After that, extraction of DNA and Polymerase Chain Reaction (PCR) were performed to amplify the target genes.

**Results:**

Twelve samples were collected from six males and six females Egyptian patients with cancer with an age range from 22 to 65 years. These cases are scarce and samples were collected over a period of almost eleven months. All isolates were confirmed as positive *H. pylori* through *colony* morphology and biochemical tests. The most effective antibiotic found was ciprofloxacin whereas all isolates showed resistance to metronidazole and erythromycin. The target CagA oncogene gene with expected product size was reported and seven (out of twelve) isolates (58%) were identified as CagA positive.

**Conclusion:**

The current study is unique in two main aspects. First, it reported the pattern of antimicrobial susceptibility and prevalence of CagA gene in *H. pylori* from Egyptian patients. Second, it exclusively recruited isolates from gastric cancer patients which were confirmed by clinical and laparoscopic examination. The moderately high prevalence of CagA gene in Egyptian cancer patients calls for more vigilance against that oncogene.

## Background

*Helicobacter .pylori (H.pylori)* is a global issue with increasing rates of infection making it one of the most damaging human pathogens [1, 2]. Infection caused by *H. pylori leads to* different gastrointestinal disorders including gastritis, gastric ulcer and gastric cancer [3, 4]. However, *H. pylori* is considered a poor man’s gut pathogen [3] because it has been mainly reported in isolates from developing countries [1, 2, 5]. Recently, World Health Organization (WHO) has classified *H. pylori* as Class I Carcinogen and a risk factor for gastric cancer, which is acknowledged worldwide as 2nd highest cause of deaths related to cancer [6, 7]. The detection of *H. pylori* is essential and therefore different types of tests are performed to identify *H. pylori* in clinical samples. Some tests, that involve an endoscopy to have biopsy samples like urease testing, bacterial culture and PCR [6, 8, 9] have shown excellent sensitivity and specificity for preliminary detection among adults.

One of the key genes of *H. pylori* is Cytotoxin-associated gene A (Cag A). The translocation of the CagA is encoded by Type IV Secretion System (T4SS) and is associated with gastric cancer [10, 11]. This relationship between H *pylori* and gastric cancer was interpreted by the injection of CagA protein into epithelial cells through T4SS system, which binds to several cellular proteins and leads to dysregulation of cell division and carcinogenesis [12].

*H. pylori* is mainly treated through proton pump inhibitors (PPIs) and antimicrobial agents such as amoxicillin, metronidazole or erythromycin. But the ever-increasing resistance against antibiotics reduces the effectiveness of any treatment involving these therapies [13]. *H. pylori* has a variable antimicrobial sensitivity pattern, depending on the geographical area and the occurrence of *H. pylori* resistance that reduces the success of first line treatment. Many studies have been reported on *H. pylori* isolates from different parts of the worlds, including Germany [14], India [15], Brazil [16, 17], Venezuela [18], Chile [19], Colombia [20], Iran [21], Indonesia [22], and Pakistan [23]. In Arabian isolates, limited reports have been published on the antimicrobial resistance and virulence factors of *H. pylori* in UAE [24] and Kuwait [25, 26]. Though the Egyptian population represents almost one fourth of the Arab nations, few studies have documented the genetic profile and antibiotic sensitivity of *H. pylori* from Egyptian isolates.

In Egypt, a high prevalence of *H. pylori* infections has been reported, ranging from 70% in the general population [27], 73% among school children [28], up to 88% in patients with chronic active HCV [29]. With respect to genotypes, CagA positive *H. pylori* strains in Egyptian isolates was not only associated with gastritis [30], gastric cancer [31], but was reported as a risk factor for ischemic heart diseases. [32]. Studies have also reported contradicting findings with respect to resistance of *H. pylori* to various antimicrobial protocols in Egyptian hospitals. For instance, resistance to Metronidazole ranged from 25% [33] to 100% [34].

The aim of the present study was two-fold as it examined: (1) The pattern of antimicrobial resistance of *H. pylori* isolates among gastric cancer patients, and (2) the prevalence of onco-protien CagA gene in isolates by PCR in Egypt.

## Methods

### Sample collection

This study was conducted in the period from November 2014 to September 2015 at Kasr El-Aini Faculty of Medicine, Cairo University, Egypt. Written consent was obtained from all participants. Gastric biopsy specimens were collected under aseptic conditions and were kept in selective tryptic soy broth as transport media. They were then preserved in the laboratory of microbiology for further processing, as recommended by Siu and colleagues [35].

### Patients’ inclusion and exclusion criteria

As per the aim of the study, we recruited only those patients who were diagnosed with gastric cancer and were positive for *H. pylori*, based on laparoscopic and clinical examination. The patients who had other primary malignancies, or had received proton pump inhibitors or antimicrobial treatment for eradication of *H. pylori* over the previous three months were excluded from the study.

### Isolation of *H. pylori*

We followed the protocol of Yamaoka and colleagues [36] for the isolation of *H. pylori,* using Columbia blood agar supplemented with 10% horse blood, and selective supplement of *H. pylori* (Dent supplement, Oxoid, UK). Then, we incubated the inoculated plates for 3 to 5 days at 37 °C under microaerophilic condition without catalyst using Campylobacter gas kit (Oxoid, UK). According to Oskouei and colleagues [37].

### Identification of *H. pylori*

We followed the protocol of Owen and his colleagues [38] in using the gold standard for identifying the salient cultural characteristics of *H. pylori*, such as morphology of colonies including shape, texture, margin and size. All slides were further microscopically examined for red curved and straight rods. The *H. pylori* was identified by its biochemical profile, according to Yamaoka and colleagues [36], such as oxidase, catalase and urease reactions.

### Storage of strains

Cultures were stored in a deep freezer at − 80 °C in a sterile Brain Heart Infusion (BHI) (Oxoid - UK), supplemented with 20% glycerol (Sigma Chemical Co. - UK).

### Antimicrobial susceptibility pattern

The antimicrobial susceptibility of *H. pylori* isolates was examined using disc diffusion method against five antimicrobial agents namely; amoxicillin (10 μg), metronidazole (5 μg), tetracycline (30 μg), ciprofloxacin (5 μg) and erythromycin (10 μg). Under microaerophilic condition, the antimicrobial discs were aseptically placed on the dried surface of Muller-Hinton’s agar (MHA) (Oxoid, UK) with 10% horse blood incubated at 37 °C for 72 h. Antimicrobial susceptibility testing to determine zones of inhibition was conformed to the standard of the Clinical and Laboratory Standard Institute (CLSI) with little modification according to previous studies with similar methodology, where a zone size < 25 mm was evaluated as resistant for amoxicillin, > 16 mm for metronidazole resistance [39], > 30 mm for tetracycline, > 17 mm for ciprofloxacin [40] and > 19 mm for erythromycin resistance [41].

### DNA extraction and PCR amplification

Extraction of DNA from *H. pylori* isolates was done from freshly harvested bacterial cells. The DNA was extracted using Qiagen’s QIAamp DNA Mini Kit (Qiagen, Germany) according to manufacturer specifications. Next, all samples were tested by glmM gene to verify *H. pylori* strains. The screening of CagA was performed by a reaction mixture that contained 1 μL of primer, 1 μL of genomic DNA, 12.5 μL PCR MasterMix, and ddH_2_O to a total volume of 25 μL. The protocol of PCR was performed starting with 5 min initial denaturation at 95 °C, 30 cycles of 30 s at 94 °C, 30 s at 52 °C, 30 s at 72 °C and a final extension of 72 °C for 5 min. PCR product was detected by gel electrophoresis. The target genes used in the study are listed in Table 1.

**Table 1:** Target gene, sequence and expected product size

Primers	Sequence (5′ → 3′)	Product size	References
glmM-F	GCATTCACAAACTTATCCCCAATC	140 bp	Espinoza et al. (2011) [55]
glmM-R	GGATAAGCTTTTAGGGGTGTTAGGGG		
CagA- F	AATACACCAACGCCTCCAAG	496 bp	Izadi et al. (2012) [56]
CagA-R	ATCTCAAGCTAACAGCCAAAA		

Extracted DNA from colonies of *H. pylori* ATCC 43504 was used as a positive control, while distilled water served as a negative one.

## Results

**Fig. 1:**
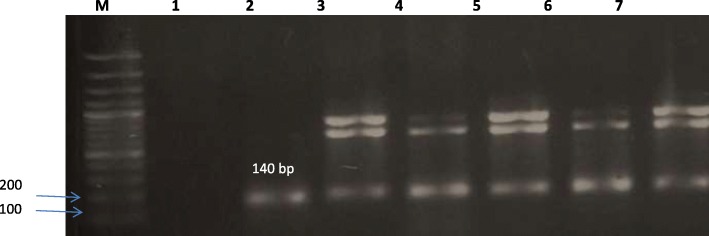
Amplification of glmM gene as a confirmatory identification of *H. pylori* isolates. (Lane M, 100 bp ladder; lane 1 negative control; lane 2 positive control)

A total of twelve samples (six males and six females) were collected from patients with an age range of 22 to 65 years as per the selection criteria. All isolates showed positive biochemical traits of *H. pylori*. The twelve isolates were confirmed as *H. pylori* by amplification of glmM gene using PCR, as demonstrated in Fig. 1.

With respect to antimicrobial sensitivity of *H. pylori* isolates of Egyptian cancer patients, as listed in Table 2, the most potent antibiotic tested was ciprofloxacin, 10 isolates out of 12 (83% sensitive), the second effective antibiotic was tetracycline, 9 out of 12 (75%), whereas one isolate (8%) was sensitive to amoxicillin. All the isolated strains were resistant to metronidazole and erythromycin.

**Table 2:** Antimicrobial susceptibility testing of *H. pylori* isolates from patient with gastric cancer by the disc diffusion method

Antimicrobial agents	Conc.	Code	Resistant isolates Number (%)	Sensitive isolates Number (%)	References
Amoxicillin	10μg	AM	11 (92%)	1 (8%)	Ogata et al. [39]
Metronidazole	5μg	MTZ	12 (100%)	0 (0%)	
Tetracycline	30μg	TE	3 (25%)	9 (75%)	Ozbey et al. [40]
Ciprofloxacin	5μg	CIP	2 (17%)	10 (83%)	Tanih et al. [41]
Erythromycin	10μg	E	12 (100%)	0 (0%)	

**Fig. 2:**
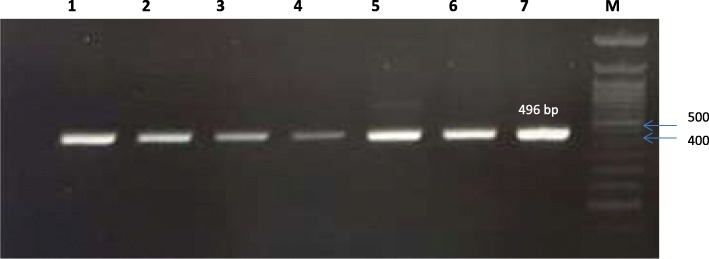
Gel electrophoresis showing that seven out of twelve of *H. pylori* isolates were Cag A positive. (Lane M, 50 bp ladder)

With respect to the second aim of the study, the prevalence of onco-protien CagA gene in the isolates was confirmed by PCR, as described in the Methodology section. Figure 1 demonstrates that seven (out of twelve) isolates (58%) were identified as CagA positive Fig. 2.

## Discussion

The current study aimed to report the antimicrobial sensitivity in *H. pylori* isolates from Egyptian patients with gastric cancer. Almost all isolates were sensitive to ciprofloxacin and tetracycline and they were resistant to metronidazole and erythromycin. These findings are consistent with those reported by Fathi and colleagues with resistance rates of 100 and 25% for metronidazole and ciprofloxacin respectively in Egypt [34]. In the same vein, amoxicillin and tetracycline were the best options in treating Egyptian patients with *H. pylori* with an excellent susceptibility of 91 and 82% respectively [42].

In other Middle Eastern countries, resistance to metronidazole in isolates of *H. pylori* was 78% [43], 57% [44] and 62% [45] in Saudi Arabia, Bahrain and United Arab Emirates (UAE), respectively. While only 2% of isolates were resistant to tetracycline in Saudi Arabia [43], almost no resistance was reported to tetracycline in Bahrain or UAE [44, 45]. A systemic review from Iran reported a growing antimicrobial resistance of *H. pylori,* particularly against metronidazole (62%), amoxicillin (16%), erythromycin (15%) and tetracycline (12%) [46, 47]. Rasheed and his colleagues reported a similar antibiotic sensitivity profile in *H. pylori* isolates from Pakistani patients, with a relatively low resistance percentages of ciprofloxacin and tetracycline, at 13 and 4% respectively and a high resistance to metronidazole and clarithromycin, at 74 and 48% correspondingly. The alarming sign was that almost 93% of Pakistani isolates showed resistance to one or more antibiotics.

As evident in literature that the *H. pylori* antibiotic resistance against metronidazole and clarithromycin is principally challenging, other treatment approaches have been suggested, which are still under investigation. These include complementary probiotic therapy with Lactobacillus that could be feasible alternate eradication therapy [48].

The second aim of the study is related to explore the prevalence of CagA gene in isolates by PCR in Egypt. The notorious reputation of CagA gene as an oncogenic protein was echoed by the results of the study, where almost 60% of isolates from gastric cancer patients were positive CagA. This is relatively higher than the prevalence of CagA, as reported by other studies, such as 46% [49], and 50% [50] in Egyptian isolates of *H. pylori.* While in other countries in the Middle East, CagA gene was even lower in *H. pylori* isolates from Kuwait (41%) [51] and Jordan (26%) [52]. The moderately high prevalence of CagA gene in Egyptian patients with gastric cancer calls for more vigilance against this oncogene.

The correlation of CagA gene with cancer was established in *H. pylori* isolates from Turkish patients [53]. However, in South Mexico, a study reported no association between CagA genotype and gastric cancer patients [54]. The reports of different studies across various geographical regions reinforce the unpredictability of the expression of CagA gene of H *Pylori* across different populations. For instance, the CagA gene showed strong signatures in isolates from Venezuelan (Amer-Indian) populations, but not in Asian ones [12] The variability of adaptation models of CagA gene in different ethnic groups shows the effect of host genetics or other lifestyle patterns that moderate its expression. Generally speaking, peptic ulcer diseases were remarkably higher in patients with CagA positive H *pylori* strains than the ones with CagA negative strains, but the presence of CagA gene has not been associated with severity [26]. Still there is a need to explore further the moderating correlation and presence of CagA gene in gastric cancer patients.

### Salient features of the study

The current study is unique in two main aspects. First, it reported the pattern of antimicrobial susceptibility and prevalence of CagA gene in *H. pylori* among Egyptian patients. Second, it exclusively recruited gastric cancer patients who had been confirmed by clinical and laparoscopic examination. As these cases are scarce, they were collected over a period of almost eleven months.

### Venues for further research

There’s a need to perform DNA sequencing of the same fragment obtained from each isolate with relevance to their antibiotic resistance pattern to confirm the results and explore the mechanism of antibiotic resistance in Egyptian isolates of *H. pylori.* A comparison can then be reported between isolates from different geographical locations, particularly in the Middle Eastern region. Moreover, regarding the correlation of CagA gene with gastric cancer, further research is needed to explore its mechanism and variables that moderate its effect in the host. This approach might facilitate to gain insight into the profile of antibiotic resistance pattern and CagA gene in Egyptian isolates of *H. pylori.*

## Conclusion

The current study reported high susceptibility of *H. pylori* to ciprofloxacin and tetracycline, which is promising to eradicate the infection in Egypt. The pattern of antimicrobial susceptibility among gastric cancer patients has to be frequently investigated to guide clinicians to choose effective antibiotics for *H. pylori* infections and monitor the antibiotics policy. The results show that CagA gene was present in almost 60% of *H. pylori* isolates from cancer patients. Therefore, it is necessary to screen it in all cases with *H. pylori* infection as its association with threating prognosis calls for more vigilance against this oncogene.

## Abbreviations

BHI: Brain Heart Infusion
CagA: Cytotoxin-associated gene A
CLSI: Clinical & Laboratory Standards Institute
MHA: Muller-Hinton’s agar
PCR: Polymerase Chain Reaction
T4SS: Type IV Secretion System
WHO: World Health Organization
